# Microbial nitrogen dynamics in organic and mineral soil horizons along a latitudinal transect in western Siberia

**DOI:** 10.1002/2015GB005084

**Published:** 2015-05-12

**Authors:** Birgit Wild, Jörg Schnecker, Anna Knoltsch, Mounir Takriti, Maria Mooshammer, Norman Gentsch, Robert Mikutta, Ricardo J Eloy Alves, Antje Gittel, Nikolay Lashchinskiy, Andreas Richter

**Affiliations:** 1 Department of Microbiology and Ecosystem Science, University of ViennaVienna, Austria; 2 Austrian Polar Research InstituteVienna, Austria; 3 Department of Earth Sciences, University of GothenburgGothenburg, Sweden; 4 Institute of Soil Science, Leibniz Universität HannoverHannover, Germany; 5 Department of Ecogenomics and Systems Biology, University of ViennaVienna, Austria; 6 Department of Biology, Centre for Geobiology, University of BergenBergen, Norway; 7 Department of Bioscience, Center for GeomicrobiologyAarhus, Denmark; 8 Central Siberian Botanical Garden, Siberian Branch of Russian Academy of SciencesNovosibirsk, Russia

**Keywords:** tundra, permafrost, boreal forest, protein depolymerization

## Abstract

Soil N availability is constrained by the breakdown of N-containing polymers such as proteins to oligopeptides and amino acids that can be taken up by plants and microorganisms. Excess N is released from microbial cells as ammonium (N mineralization), which in turn can serve as substrate for nitrification. According to stoichiometric theory, N mineralization and nitrification are expected to increase in relation to protein depolymerization with decreasing N limitation, and thus from higher to lower latitudes and from topsoils to subsoils. To test these hypotheses, we compared gross rates of protein depolymerization, N mineralization and nitrification (determined using ^15^N pool dilution assays) in organic topsoil, mineral topsoil, and mineral subsoil of seven ecosystems along a latitudinal transect in western Siberia, from tundra (67°N) to steppe (54°N). The investigated ecosystems differed strongly in N transformation rates, with highest protein depolymerization and N mineralization rates in middle and southern taiga. All N transformation rates decreased with soil depth following the decrease in organic matter content. Related to protein depolymerization, N mineralization and nitrification were significantly higher in mineral than in organic horizons, supporting a decrease in microbial N limitation with depth. In contrast, we did not find indications for a decrease in microbial N limitation from arctic to temperate ecosystems along the transect. Our findings thus challenge the perception of ubiquitous N limitation at high latitudes, but suggest a transition from N to C limitation of microorganisms with soil depth, even in high-latitude systems such as tundra and boreal forest.

**Key Points:**

1. We compared soil N dynamics of seven ecosystems along a latitudinal transect
2. Shifts in N dynamics suggest a decrease in microbial N limitation with depth
3. We found no decrease in microbial N limitation from arctic to temperate zones

## 1 Introduction

Protein depolymerization is considered the bottleneck for soil N cycling [*Schimel and Bennett*, 2004], given that a large proportion of soil N is bound in proteins [*Jones and Kielland*, 2002], but can only be used by plants and microorganisms after depolymerization by extracellular enzymes. Nitrogen made available through protein depolymerization (i.e., oligopeptides and amino acids) is rapidly taken up by soil microorganisms [e.g., *Jones and Kielland*, 2002; *Kielland et al.*, 2007; *Hill et al.*, 2012; *Farrell et al.*, 2013] and used to form new biomass. If N uptake exceeds the microbial N demand, microorganisms release excess N as ammonium (N mineralization), which in turn can serve as substrate for nitrification [*Schimel and Bennett*, 2004]. The balance between protein depolymerization, N mineralization, and nitrification thus reflects the degree of microbial N limitation.

Nitrogen mineralization and nitrification have been the focus of numerous studies (for a metaanalysis, see *Booth et al.* [2005]), whereas gross rates of protein depolymerization have only been reported in four studies investigating decomposing beech leaf litter [*Wanek et al.*, 2010; *Mooshammer et al.*, 2012], arctic permafrost soils [*Wild et al.*, 2013], and a biochar-amended agricultural soil [*Prommer et al.*, 2014]. So far, the variability of protein depolymerization rates, and possible controls on these rates, remain unknown. Furthermore, although protein depolymerization is considered to constrain soil N cycling, empirical evidence linking protein depolymerization rates to microbial N limitation, as well as to other N transformations such as N mineralization and nitrification in soils is scarce.

Microbial N limitation results from an imbalance between N supply and N demand and has been related to differences in the elemental compositions of microorganisms and their substrate. As suggested by the theory of ecological stoichiometry, N limitation of microbial growth is expected where the C/N ratio of organic matter, after subtracting the C costs for microbial maintenance respiration [*Sinsabaugh et al.*, 2013], exceeds the C/N ratio of microbial biomass, i.e., where microorganisms face an excess of C over N in relation to their biomass [*Sterner and Elser*, 2002; *Mooshammer et al.*, 2014b].

C/N ratios of organic matter are highly variable. For instance, C/N ratios are high in fresh plant litter, but decrease during decomposition, since C is lost at higher rates than N [*Sterner and Elser*, 2002; *Mooshammer et al.*, 2014b]. C/N ratios decline from litter layer over organic to mineral soil horizons, as organic matter becomes increasingly decomposed with soil depth [*Rumpel and Kögel-Knabner*, 2011]. As a consequence, also microbial N limitation is expected to decrease with depth.

C/N ratios of organic matter also differ between ecosystems, with higher C/N in high-latitude systems such as tundra or boreal forest [*Post et al.*, 1985; *X. Xu et al.*, 2013]. In these ecosystems, the decomposition of organic matter is constrained by low litter quality and low activity of microbial decomposers [*Hobbie et al.*, 2000]. Low decomposition rates of organic matter, together with its high C/N ratios, have been held responsible for N limitation of plant and microbial growth in tundra and boreal forest [*Vitousek and Howarth*, 1991], which has been frequently observed [e.g., *Shaver and Chapin*, 1980; *Hobbie et al.*, 2002; *Sistla et al.*, 2012]. As conditions become more favorable for decomposition toward lower latitudes, microbial N limitation is also expected to decrease [*Schimel and Bennett*, 2004].

The objective of this study was therefore to test the connection between soil C/N ratios and microbial N transformations and to dissect the effects of ecosystem type and soil depth. We hypothesized that following changes in the stoichiometric imbalance between microorganisms and their substrate, N limitation would decrease from arctic over boreal to temperate ecosystems within the organic topsoil and from organic topsoil to mineral subsoil within each of the ecosystems, resulting in a higher allocation of N to mineralization and nitrification. To test these hypotheses, we established a 1500 km latitudinal transect in western Siberia, from 67°N (tundra) to 54°N (steppe), corresponding to a range in mean annual temperature from −7.1 to 1.0°C. We selected seven sites along this transect that were characterized by zonal vegetation and low anthropogenic influence. At each site, we sampled three soil horizons (grouped into organic topsoil, mineral topsoil, and mineral subsoil) and measured C/N ratios of soil organic matter (SOM), the extractable soil fraction, and the microbial biomass, as well as gross rates of protein depolymerization, N mineralization, and nitrification using a set of ^15^N pool dilution assays.

## 2 Material and Methods

### 2.1 Sampling Sites

**Figure 1:**
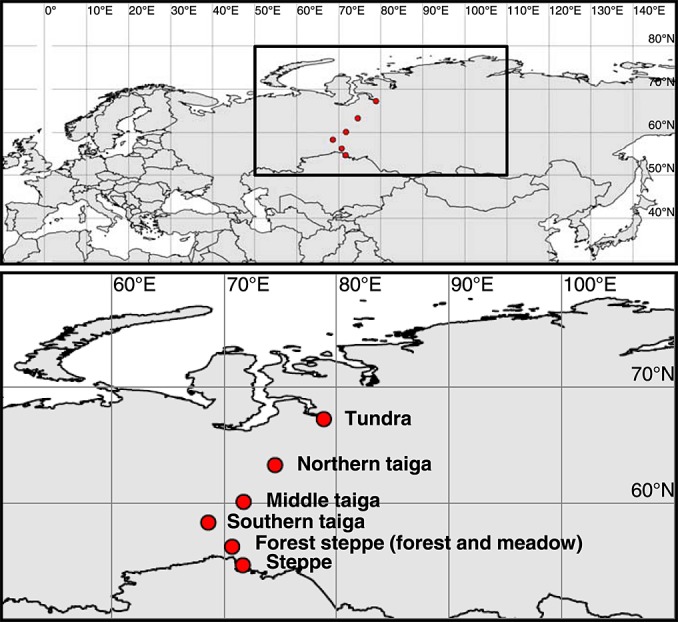
Map of sampling sites along a latitudinal transect in western Siberia.

Soil samples were taken from seven ecosystems along a 1500 km latitudinal transect in western Siberia: Tundra, northern taiga, middle taiga, and southern taiga, forest steppe (two sites, see below), and steppe (Figure 1; map created using the cshapes package of *R* [*Weidmann et al.*, 2010; *R Development Core Team*, 2012]). Forest steppe is a dominant land cover type in the temperate south of Siberia, characterized by patches of deciduous forest mixed with grassland. We sampled both forest and grassland sites and further refer to them as “Forest steppe: Forest” and “Forest steppe: Meadow.” For details on the sampling sites, see Table1. Climate data were derived from *Stolbovoi and McCallum* [2002], soil classification follows the World Reference Base for Soil Resources [*IUSS Working Group World Reference Base*, 2007].

**Table 1:** Basic Characterization of Sites Along the Latitudinal Transect

						Organic Topsoil^b^		Mineral Topsoil^b^		Mineral Subsoil^b^	
	Coordinates	Mean Annual Temperature (MAT)^a^	Mean Annual Precipitation (MAP)^a^	Dominant Plant Species	Soil Type	Horizon	Depth	Horizon	Depth	Horizon	Depth
Tundra	67°16′N 78°50′E	−7.6	392	*Betula nana*, *Cladonia* spp.	Turbic Cryosol	O	0–6	A	2–13	Bg, BCg	6–57
Northern taiga	63°17′N 74°32′E	−4.6	430	*Picea obovata*, *Larix sibirica*	Histic Podzol	Oi, Oe	0–22	AE, EA	8–30	Bg	14–47
Middle taiga	60°09′N 71°43′E	−2.2	438	*Abies sibirica*, *Picea obovata*	Endogleyic Regosol	Oi	0– 6	A, AE, EA	6–14	E, EA	12–55
Southern taiga	58°18′N 68°35′E	−0.5	396	*Picea obovata*, *Abies sibirica*	Albic Podzol	Oi	0–7	A, AE	4–18	E, EA	15–59
Forest steppe: Forest	56°14′N 70°43′E	0.7	340	*Populus tremula*, *Betula pendula*	Haplic Phaeozem	O, Oa	0–10	A	7–46	B	57–109
Forest steppe: Meadow	56°14′N 70°43′E	0.7	340	*Calamagrostis epigeios*, *C. arundinacea*	Luvic Phaeozem	Oa	0–7	A	4–35	Bt	26–84
Steppe	54°41′N 71°38′E	1.0	309	*Stipa capillata*, *Festuca valesiaca*	Calcic Kastanozem	OA	0–12	Ak	8–37	Bk	27–109

a MAT: mean annual temperature (in °C); MAP: mean annual precipitation (in mm).

b Horizon description and sampling depth (in cm) for organic topsoil, mineral topsoil, and mineral subsoil sampled in five replicate soil pits at each site.

Soils were sampled in August 2012 traveling from north (tundra) to south (steppe); i.e., soils were sampled in the late growing season at the respective site. At all sites, we sampled the three dominant soil horizons of five replicate soil pits, in total amounting to 105 soil samples from 35 pedons. We further refer to the first horizons (O and OA) as organic topsoil, to the second (A, AE, or EA) as mineral topsoil, and to the third (E, B, or BC) as mineral subsoil. The category of organic topsoil thus also includes the uppermost horizons of the steppe site that technically qualified as mineral horizons due to a comparatively low C content; we specify where these horizons deviate from organic topsoil horizons of other sites. Sampled soil horizons, as well as sampling depths are listed in Table1. Plant roots were carefully removed, and samples sieved to 2 mm, except for the tundra, where samples were too moist for sieving and were homogenized by hand. Before further processing, soil water content was adjusted to a minimum of 60% (organic topsoil, except steppe), 15% (mineral topsoil, plus the steppe top horizon), or 10% (mineral subsoil) with deionized water.

### 2.2 Carbon and Nitrogen Pools

Organic C and total N content were determined in dried (60°C) and ground samples with Elemental Analysis–Isotope Ratio Mass Spectrometry (EA-IRMS) (CE Instrument EA 1110 elemental analyzer, coupled to a Finnigan MAT DeltaPlus IRMS with a Finnigan MAT ConFlo II Interface). Mineral topsoil and subsoil at both forest steppe sites, as well as all horizons of the steppe site, contained carbonate. Carbonate was removed from these samples by acidification with HCl following *Prommer et al.* [2014] before EA-IRMS analysis.

Total dissolved N was measured in 1 M KCl extracts with a Dissolved Organic Carbon/Total Nitrogen (DOC/TN) analyzer (Shimadzu TOC-V_CPH/CPN_/TNM-1). Ammonium and nitrate concentrations were determined photometrically in 1 M KCl extracts following *Kandeler and Gerber* [1988] and *Miranda et al.* [2001], respectively; and total free amino acids were quantified fluorometrically using the o-phthaldialdehyde and β-mercaptoethanol method [*Jones et al.*, 2002]. Dissolved organic N was calculated by subtracting ammonium and nitrate from total dissolved N concentrations. Soil extracts with 1 M KCl were also used to determine pH values.

Microbial C and N were estimated using chloroform-fumigation-extraction [*Kaiser et al.*, 2011]: Soil samples fumigated with chloroform, as well as unfumigated samples, were extracted with 0.5 M K_2_SO_4_. Dissolved organic C and total dissolved N were determined in both sets of extracts with DOC/TN analysis, and microbial C and N were calculated as the difference between fumigated and nonfumigated samples. Carbon and N released from microbial cells by chloroform fumigation are mostly cytoplasmic; since correction factors reported in the literature are highly variable [e.g., *Brookes et al.*, 1985, and references therein], we present these data as they were measured, without applying correction factors. C/N ratios of SOM (C/N_SOM_), K_2_SO_4_ extracts (C/N_extr_), and microbial biomass (C/N_mic_) were calculated as mass ratios, and stoichiometric imbalances were calculated as the ratios of C/N_SOM_ or C/N_extr_ over C/N_mic_. Corresponding data on microbial community composition and extracellular enzyme activities were published by *Schnecker et al.* [2015].

### 2.3 Gross Nitrogen Transformation Rates

Gross rates of protein depolymerization, N mineralization, and nitrification, as well as the corresponding microbial uptake rates of amino acids, ammonium, and nitrate, were measured using ^15^N pool dilution assays, by labeling the respective product pool (amino acids, ammonium, or nitrate) with ^15^N and calculating the fluxes into and out of the pool from the dilution of ^15^N between two time points.

Gross rates of protein depolymerization and microbial amino acid uptake were determined following *Wanek et al.* [2010], with the modifications for soil samples described by *Wild et al.* [2013]. Duplicates of fresh soil (organic topsoil: 1 g, mineral topsoil: 2 g, and mineral subsoil: 4 g) were amended with either 1 mL (organic topsoil) or 0.5 mL (mineral topsoil and subsoil) of a 2.5 µg mL^−1^ solution of ^15^N labeled amino acids (mixture of 20 amino acids of >98 at% ^15^N, Spectra and Cambridge Isotope Laboratories, dissolved in 10 mM CaSO_4_), corresponding to between 0.3 and 10.2 µg amino acids per gram dry soil, depending on soil horizon. Soil samples were incubated at 15°C, which is in the range of average air temperatures in August along the studied transect [*Stolbovoi and McCallum*, 2002]. Duplicates were extracted after 10 or 30 min, respectively, with 20 mL 10 mM CaSO_4_ containing 3.7% formaldehyde to stop microbial activity. Samples were centrifuged for 5 min at 10,845 g, filtered through synthetic wool and GF/C filters (Whatman) and loaded on precleaned cation exchange cartridges (OnGuard II H 1cc cartridges, Dionex). Blanks and amino acid standards were processed with the samples. Amino acids were eluted from the cartridges with 10 mL 3 M NH_3_, and an internal standard (1 µg nor-valine, nor-leucine, and para-chloro-phenylalanine each, Sigma-Aldrich) was added. Samples were dried under N_2_, redissolved in 20% ethanol, and redried in a SpeedVac system. Amino acids were derivatized with ethyl-chloroformate [*Wanek et al.*, 2010] before analysis with gas chromatography-mass spectrometry (Thermo TriPlus Autosampler, Trace GC Ultra and ISQ mass spectrometer, with an Agilent DB-5 column; PTV injection in splitless mode at 270°C, 1 mL min^−1^ helium as carrier, temperature program: 60°C for 1.5 min, first ramp 5°C min^−1^ to 200°C, second ramp 15°C min^−1^ to 300°C, 300°C for 4 min). We calculated concentrations of alanine, aspartate + asparagine, glutamate + glutamine, isoleucine, leucine, phenylalanine, proline, and valine by calibration against external standards, and ^15^N isotopic compositions from the peak areas of amino acid fragments as described by *Wanek et al.* [2010]. Glutamine and asparagine were included in the peaks of glutamate and aspartate, respectively, since the addition of formaldehyde causes deamination of glutamine to glutamate and of asparagine to aspartate [*Wanek et al.*, 2010].

Gross rates of N mineralization and nitrification, as well as of microbial ammonium and nitrate uptake, were determined as described by *Wild et al.* [2013]. We added 0.5 mL of 0.125 mM (NH_4_)_2_SO_4_ (N mineralization and ammonium uptake) or 0.25 mM KNO_3_ (nitrification and nitrate uptake), labeled with 10% at ^15^N, to duplicates of 2 g (organic and mineral topsoil) or 4 g (mineral subsoil) fresh soil. Duplicates were incubated at 15°C for 4 h or 24 h, respectively, before extraction with 13 mL 2 M KCl and filtration through ash-free cellulose filters (Whatman 40). For N mineralization and ammonium uptake, ammonium in the extracts was isolated using acid traps and analyzed with EA-IRMS. For nitrification and nitrate uptake, nitrate in the extracts was converted to N_2_O using sodium azide under acidic conditions, following *Lachouani et al.* [2010]. Where nitrate concentrations were below the detection limit, samples were spiked with KNO_3_ of known concentration and isotopic composition before conversion to N_2_O. Derived N_2_O was analyzed with a GasBench-IRMS system (CTC Analytics autosampler, Thermo GasBench II, PreCon, and Delta V Advantage IRMS). Results from spiked samples were corrected for the contribution of added KNO_3_. All gross rates were calculated using the equations in *Kaiser et al.* [2011].

The presented gross rates are based on laboratory incubations of sieved soil samples, which is necessary to ensure a homogenous distribution of ^15^N in the soil sample and the immediate termination of microbial activity after the incubation time. However, this approach induces a certain bias against mycorrhizal fungi, and we therefore note that the contribution of mycorrhizal fungi to soil N cycling might be underestimated in this study.

### 2.4 Statistical Analysis

All statistical analyses were performed using R 2.15.0 [*R Development Core Team*, 2012] with the additional package GenABEL [*Aulchenko et al.*, 2007]. We applied one-way analysis of variance (ANOVA) followed by Tukey honest significant difference tests to assess differences between soil horizons and sites. To describe patterns of N transformations across soil horizons and sites, we calculated ratios between gross rates of protein depolymerization, N mineralization, and nitrification, and performed two-way ANOVA to compare effect sizes of horizon versus site. Data were transformed where necessary to meet conditions for ANOVA. In few cases, conditions could not be met even after transformation and Mann-Whitney-U tests were applied. To test for significant correlations, data were rank transformed before application of Pearson’s correlation, thus effectively testing for a monotonous relationship. For additive effects of C and N content, as well as of microbial C and N on gross rates of N transformations, we additionally performed a multiple regression on rank-transformed data. Differences and correlations were considered significant at *p* < 0.05.

## 3 Results

### 3.1 Soil Organic Matter Content, Microbial Biomass, and pH

In tundra, taiga, and forest steppe systems, organic C content in the organic topsoil ranged between 20% and 45% of soil dry weight, with highest C contents in northern and middle taiga (Table2), whereas in the steppe, the uppermost horizon contained only 4% of organic C (see above). At all sites, organic C content decreased significantly with soil depth (Table2 and Table S1 in the supporting information), accounting for 2–7% in the mineral topsoil and less than 2% in the mineral subsoil. The total N content of SOM, as well as microbial C and N, was strongly correlated with organic C content (in all cases, *p* < 0.001 and *R*^2^ > 0.78) and also decreased in concentration with soil depth.

**Table 2:** Basic Characterization of Sampled Soil Horizons^a^

	C (mg g^−1^ d.s.)		N (mg g^−1^ d.s.)		Microbial C (µg g^−1^ d.s.)		Microbial N (µg g^−1^ d.s.)		C/N_SOM_^b^		C/N_extr_^b^		C/N_mic_^b^		Imbalance_SOM_^b^		Imbalance_extr_^b^		pH	
Tundra																				
Organic topsoil	307.9	(37.4)	8.8	(0.7)	2288.8	(364.6)	328.0	(40.4)	34.9	(3.5)	21.0	(1.5)	6.9	(0.3)	5.1	(0.5)	3.0	(0.1)	3.8	(0.1)
Mineral topsoil	30.4	(3.0)	1.8	(0.1)	290.3	(54.5)	30.5	(5.5)	16.4	(0.7)	19.1	(3.0)	9.5	(0.3)	1.7	(0.1)	2.0	(0.3)	3.7	(0.0)
Mineral subsoil	4.1	(0.5)	0.4	(0.0)	29.1	(6.1)	1.7	(0.3)	11.1	(0.6)	32.1	(8.7)	18.6	(3.2)	0.7	(0.1)	2.4	(0.7)	3.9	(0.1)
Northern taiga																				
Organic topsoil	448.4	(7.0)	12.5	(0.3)	2132.6	(52.2)	331.7	(12.7)	35.9	(0.7)	16.9	(1.3)	6.5	(0.2)	5.6	(0.2)	2.6	(0.3)	2.8	(0.0)
Mineral topsoil	37.0	(3.1)	1.4	(0.1)	200.7	(26.4)	13.7	(1.7)	27.4	(2.0)	18.3	(2.8)	14.8	(1.3)	1.9	(0.3)	1.3	(0.2)	3.1	(0.1)
Mineral subsoil	8.2	(1.7)	0.5	(0.1)	132.8	(15.3)	3.4	(0.3)	15.7	(1.5)	33.3	(0.9)	38.7	(2.7)	0.4	(0.0)	0.9	(0.1)	3.7	(0.1)
Middle taiga																				
Organic topsoil	426.1	(24.5)	17.4	(1.0)	3669.1	(382.0)	505.4	(57.6)	24.5	(0.5)	15.0	(0.6)	7.3	(0.4)	3.4	(0.2)	2.1	(0.1)	3.7	(0.1)
Mineral topsoil	74.7	(17.3)	3.5	(0.6)	489.4	(115.9)	47.4	(13.1)	20.8	(1.8)	11.3	(0.7)	11.0	(0.9)	2.0	(0.3)	1.1	(0.1)	3.3	(0.1)
Mineral subsoil	16.7	(3.8)	1.0	(0.1)	136.0	(27.4)	5.4	(0.9)	16.3	(1.7)	22.4	(2.2)	25.2	(2.8)	0.7	(0.1)	0.9	(0.1)	3.5	(0.0)
Southern taiga																				
Organic topsoil	398.2	(18.3)	15.8	(0.9)	3064.7	(651.5)	627.7	(79.4)	25.4	(0.8)	13.3	(2.6)	4.8	(0.7)	5.8	(1.0)	3.1	(0.8)	4.3	(0.1)
Mineral topsoil	43.4	(3.6)	3.1	(0.2)	301.9	(21.9)	36.3	(3.3)	14.0	(0.8)	10.6	(0.5)	8.4	(0.6)	1.7	(0.1)	1.3	(0.0)	3.6	(0.1)
Mineral subsoil	4.8	(0.3)	0.5	(0.0)	62.2	(4.9)	3.4	(0.2)	9. 4	(0.2)	19.9	(1.0)	18.2	(0.9)	0.5	(0.0)	1.1	(0.1)	3.8	(0.1)
Forest steppe: Forest																				
Organic topsoil	292.9	(24.1)	17.7	(1.3)	2504.1	(426.5)	399.5	(66.7)	16.5	(0.3)	12.8	(0.6)	6.3	(0.4)	2.7	(0.2)	2.0	(0.1)	6.6	(0.4)
Mineral topsoil	45.6	(4.5)	3.6	(0.4)	155.8	(9.4)	11.5	(0.8)	12.9	(0.2)	10.6	(0.5)	13.6	(0.3)	0.9	(0.0)	0.8	(0.1)	4.3	(0.1)
Mineral subsoil	5.2	(0.1)	0.5	(0.0)	46.9	(1.9)	2.9	(0.1)	10.1	(0.4)	20.3	(1.1)	16.3	(0.7)	0.6	(0.0)	1.2	(0.1)	4.1	(0.0)
Forest steppe: Meadow																				
Organic topsoil	202.1	(22.7)	14.0	(1.6)	2585.0	(368.8)	390.4	(30.4)	14.4	(0.2)	12.1	(0.7)	6.5	(0.5)	2.3	(0.2)	1.9	(0.2)	5.5	(0.3)
Mineral topsoil	24.5	(1.6)	1.9	(0.1)	198.3	(20.3)	14.9	(1.6)	13.0	(0.1)	12.4	(0.5)	13.4	(0.4)	1.0	(0.0)	0.9	(0.0)	4.1	(0.0)
Mineral subsoil	5.8	(0.3)	0.5	(0.0)	53.2	(4.0)	2.7	(0.2)	10.7	(0.2)	20.3	(1.9)	19.6	(1.0)	0.6	(0.0)	1.1	(0.1)	4.0	(0.1)
Steppe																				
Organic topsoil	36.9	(3.0)	3.3	(0.3)	400.6	(73.0)	36.1	(7.4)	11.1	(0.1)	9.3	(1.0)	11.3	(0.4)	1.0	(0.0)	0.8	(0.1)	4.6	(0.1)
Mineral topsoil	20.1	(2.7)	1.8	(0.2)	247.2	(38.0)	17.9	(2.6)	10.8	(0.3)	12.0	(0.5)	13.9	(0.6)	0.8	(0.0)	0.9	(0.1)	5.1	(0.3)
Mineral subsoil	7.2	(0.8)	0.8	(0.1)	87.9	(7.1)	5.0	(0.8)	9.2	(0.2)	15.6	(0.4)	19.5	(3.0)	0.5	(0.1)	0.9	(0.1)	7.9	(0.4)

a All values are means with standard errors in brackets. For statistical analysis, see Table S1 in the supporting information. The d.s. stands for dry soil.

b C/N ratios were calculated on a mass basis. Imbalance_SOM_ was calculated as the ratio of C/N_SOM_ over C/N_mic_, Imbalance_extr_ as the ratio of C/N_extr_ over C/N_mic_.

The C/N ratio of SOM decreased from organic topsoil to mineral topsoil and further to mineral subsoil (C/N_SOM_: 23.2 ± 1.6, 16.5 ± 1.0, and 11.8 ± 0.6, respectively; means ± standard errors), whereas the C/N ratios of the extractable soil fraction and the microbial biomass increased (C/N_extr_: 14.3 ± 0.8, 13.5 ± 0.8, 23.2 ± 1.5; C/N_mic_: 7.1 ± 0.4, 12.1 ± 0.5, and 22.4 ± 1.5). This corresponds to a decrease in the stoichiometric imbalance between C/N_SOM_, but also C/N_extr_, and C/N_mic_ with depth. In the organic topsoil, C/N_SOM_ and C/N_extr_ by far exceeded C/N_mic_ (except for the steppe site), whereas in mineral topsoil and subsoil, the imbalance was significantly lower (Table2 and Table S1 in the supporting information). In the organic topsoil, we further found a decrease in C/N_SOM_ and C/N_extr_, as well as in the stoichiometric imbalances between C/N_SOM_ and C/N_extr_ over C/N_mic_ from higher to lower latitudes along the transect (Table2 and Table S2 in the supporting information).

The pH values ranged between 2.8 and 7.9, with higher values in forest steppe and steppe than in tundra and taiga (Table2). Differences in pH values between soil horizons were significant but did not show a consistent pattern (Table S1 in the supporting information).

### 3.2 Protein Depolymerization, Nitrogen Mineralization, and Nitrification

**Figure 2:**
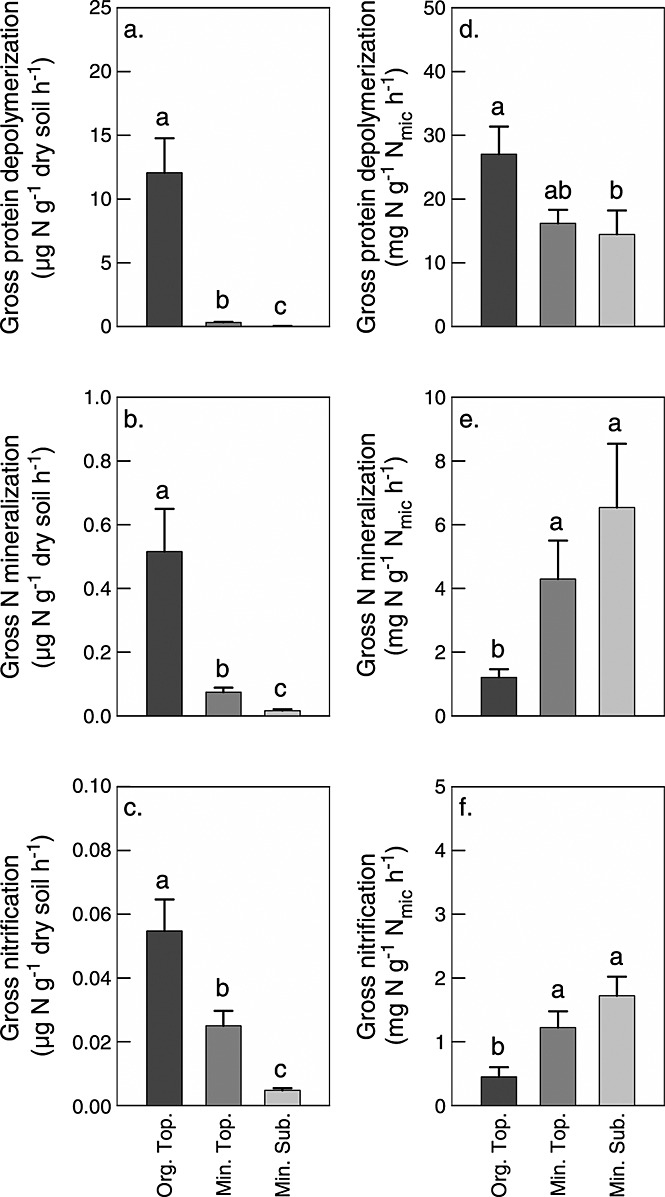
Gross rates of protein depolymerization, N mineralization, and nitrification in organic topsoil (Org. Top.), mineral topsoil (Min. Top.), and mineral subsoil (Min. Sub.), related to (a–c) dry soil and (d–f) microbial N (N_mic_). The bars represent means with standard errors across the seven ecosystems studied. The different letters indicate significant differences between horizons. Note the differences in scaling. For differences between horizons at individual sites, see Table S3 in the supporting information.

**Figure 3:**
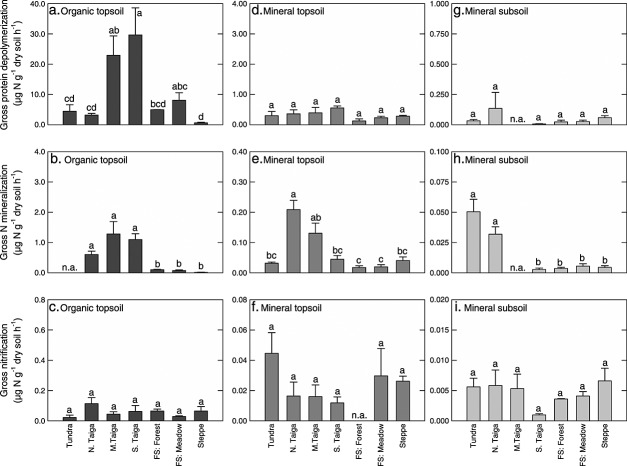
Gross rates of protein depolymerization, N mineralization, and nitrification in three soil horizons of seven ecosystems along a latitudinal transect in western Siberia. All bars represent means with standard errors. The different letters indicate significant differences between sites for each horizon. Note the differences in scaling. N. Taiga: northern taiga; M. Taiga: middle taiga; S. Taiga: southern taiga; and FS: forest steppe.

Gross rates of protein depolymerization differed by more than 2 orders of magnitude between soil horizons, with average rates of 12.07 ± 2.69, 0.33 ± 0.04, and 0.04 ± 0.01 µg N g^−1^ dry soil h^−1^ in organic topsoil, mineral topsoil, and mineral subsoil, respectively (Figure 2). Furthermore, we found significant differences in protein depolymerization rates between organic topsoil horizons of different ecosystems, with the highest rates in middle and southern taiga. In mineral topsoil and mineral subsoil horizons, we found no significant differences between ecosystems (Figure 3).

Gross N mineralization and nitrification rates were in almost all cases lower than protein depolymerization rates. Like protein depolymerization, N mineralization and nitrification decreased with soil depth, although less sharply (Figure 2). When gross rates were related to microbial N, protein depolymerization rates were within the same order of magnitude in all soil horizons and decreased only slightly with soil depth. Rates of N mineralization and nitrification, in contrast, significantly increased with soil depth (Figure 2).

All measured N transformation rates were significantly correlated with SOM C and N contents, as well as with microbial C and N (Table3). These correlations were stronger for protein depolymerization (*R*^2^ between 0.72 and 0.79) than for N mineralization (*R*^2^ between 0.34 and 0.51) and nitrification (*R*^2^ between 0.30 and 0.35), suggesting that protein depolymerization was more closely connected to properties of SOM. Altogether, SOM C and N, as well as microbial C and N, explained 82%, 58%, and 31% of the variability in protein depolymerization, N mineralization, and nitrification rates, respectively.

**Table 3:** Coefficients of Determination (*R*^2^) for Correlations of Gross Rates of Protein Depolymerization, N Mineralization, and Nitrification^a^

	Protein Depolymerization				N Mineralization				Nitrification			
	All	Org. Top.^b^	Min. Top.^b^	Min. Sub.^b^	All	Org. Top.^b^	Min. Top.^b^	Min. Sub.^b^	All	Org. Top.^b^	Min. Top.^b^	Min. Sub.^b^
C	0.716	0.147	n.s.	0.394	0.505	0.755	n.s.	n.s.	0.351	n.s.	n.s.	n.s.
N	0.789	0.564	n.s.	0.237	0.336	0.340	n.s.	0.146	0.330	n.s.	n.s.	n.s.
C_mic_^c^	0.734	0.252	0.242	n.s.	0.435	0.435	n.s.	n.s.	0.316	n.s.	n.s.	n.s.
N_mic_^c^	0.779	0.561	0.314	n.s.	0.444	0.511	n.s.	n.s.	0.302	n.s.	n.s.	n.s.
C+N+C_mic_+N_mic_	0.816	0.662	0.228	0.354	0.584	0.793	0.446	0.278	0.307	n.s.	n.s.	n.s.

a All rates and concentrations were related to dry soil and rank transformed before applying correlations. Only correlations significant at *p* < 0.05 are given (n.s. stands for not significant).

b Org. Top.: organic topsoil; Min. Top.: mineral topsoil; and Min. Sub.: mineral subsoil.

c C_mic_: microbial C; and N_mic_: microbial N.

Nitrogen mineralization and nitrification were significantly correlated with protein depolymerization and with each other (in all cases, *p* < 0.001; *R*^2^ = 0.58 for protein depolymerization versus N mineralization; *R*^2^ = 0.27 for protein depolymerization versus nitrification; *R*^2^ = 0.26 for N mineralization versus nitrification). These correlations were only significant over the range of all soil horizons. In mineral topsoil and subsoil horizons individually, we found no significant correlations between N transformation rates, whereas in organic topsoils, protein depolymerization was significantly correlated with N mineralization (*p* < 0.01, *R*^2^ = 0.34). Omitting the top horizon of the steppe site rendered the correlation not significant (*p* = 0.16, *R*^2^ = .11). Nitrification was not significantly correlated with protein depolymerization or N mineralization in either horizon. Gross rates of microbial amino acid, ammonium, and nitrate uptake closely followed the respective production rates; data are presented in Figure S1 in the supporting information.

**Figure 4:**
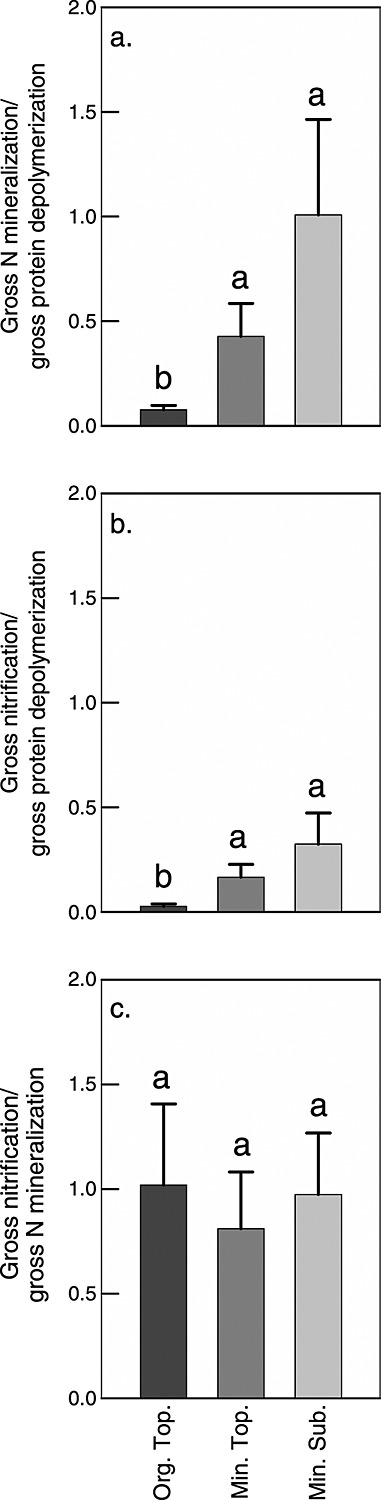
Ratios between gross rates of protein depolymerization, N mineralization, and nitrification in organic topsoil (Org. Top.), mineral topsoil (Min. Top.), and mineral subsoil (Min. Sub.). The bars represent means with standard errors across the seven ecosystems studied. The different letters indicate significant differences between horizons. For differences between horizons at individual sites, see Table S3 in the supporting information.

To describe patterns of N dynamics across sites and soil horizons, we calculated ratios between gross rates of protein depolymerization, N mineralization, and nitrification. Ratios of N mineralization over protein depolymerization and nitrification over protein depolymerization were significantly higher in the mineral topsoil and subsoil than in the organic topsoil (Figure 4). Overall, differences between horizons accounted for 36% and 27% of the variability in ratios of N mineralization or nitrification over protein depolymerization, respectively, and differences between sites for 21% and 25% (in all cases, *p* < 0.01, two-way ANOVA). Interactive effects were not significant. For the ratio of nitrification over N mineralization, differences between sites accounted for 30% of the variability, and interactive effects between sites and horizons for 33% (for both *p* < 0.01). Effects of horizons alone were not significant due to high ratios in the top horizon of the steppe site. Removing these data resulted in a pattern similar to the other ratios, with significantly higher ratios in the mineral subsoil than in the organic topsoil.

**Figure 5:**
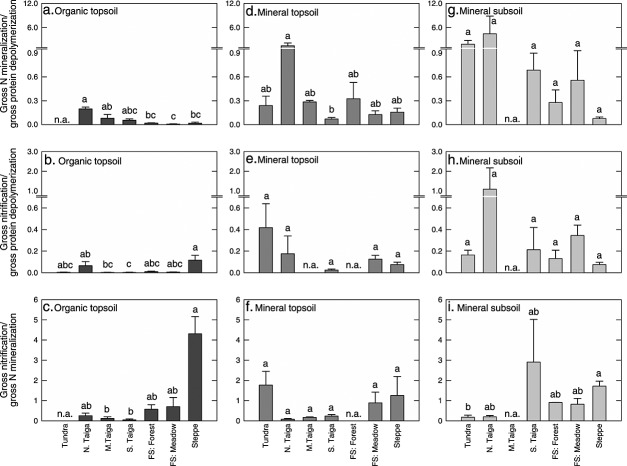
Ratios between gross rates of protein depolymerization, N mineralization, and nitrification in three soil horizons of seven ecosystems along a latitudinal transect in western Siberia. All bars represent means with standard errors. The different letters indicate significant differences between sites for each horizon. N. Taiga: northern taiga; M. Taiga: middle taiga; S. Taiga: southern taiga; and FS: forest steppe.

In contrast to the patterns between horizons, we did not find consistent patterns between ecosystems that would point at a systematic change in soil N dynamics along the latitudinal transect (Figure 5). For the organic topsoil, N mineralization and nitrification were consistently low compared to protein depolymerization, whereas in mineral topsoil and subsoil, ratios were highly variable even within ecosystems, supporting the observed decoupling between individual N transformation processes in mineral soil horizons. Furthermore, all calculated ratios between protein depolymerization, N mineralization, and nitrification were significantly correlated with stoichiometric imbalances between the C/N ratios of SOM or the extractable soil fraction and the C/N ratio of the microbial biomass, although correlations were rather weak (*R*^2^ between 0.06 and 0.27; Table4).

**Table 4:** Coefficients of Determination (*R*^2^) for Correlations Between Stoichiometric Imbalances and Ratios Between N Transformation Rates[Table-fn tf4-1]

	Imbalance_SOM_	Imbalance_extr_
Gross N mineralization/gross protein depolymerization	0.063	n.s.
Gross nitrification/gross protein depolymerization	0.242	0.132
Gross nitrification/gross N mineralization	0.196	0.265

a All values were rank transformed before applying correlations. Only correlations significant at *p* < 0.05 are given (n.s. stands for not significant).

b Imbalance_SOM_ was calculated as the ratio of C/N_SOM_ over C/N_mic_, Imbalance_extr_ as the ratio of C/N_extr_ over C/N_mic_.

### 3.3 Nitrogen Pools

**Figure 6:**
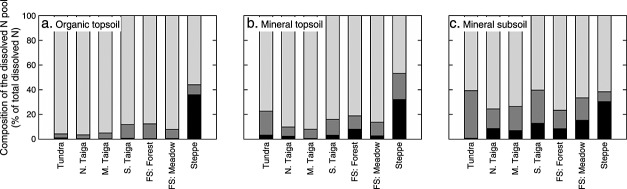
Contribution of nitrate (black), ammonium (dark grey), and dissolved organic N (light grey) to the total dissolved N pool in three soil horizons of seven ecosystems along a latitudinal transect in western Siberia. N. Taiga: northern taiga; M. Taiga: middle taiga; S. Taiga: southern taiga; and FS: forest steppe. For absolute concentrations of N pools, see Table S4 in the supporting information; for statistical analysis, see Table S5 in the supporting information.

In organic topsoils of tundra, taiga, and forest steppe, the pool of dissolved N was dominated by organic N forms (88–97% of total dissolved N), of which on average 12% were in the form of free amino acids (Table S4 in the supporting information). Ammonium accounted for 3–12% of total dissolved N, and nitrate for 1% or less (Figure 6). With soil depth, the contribution of organic N decreased (mineral topsoil: 77–92%; mineral subsoil: 60–77%), whereas contributions of both ammonium and nitrate increased (mineral topsoil: 7–19% ammonium, 1–8% nitrate; mineral subsoil: 15–39% ammonium, 1–15% nitrate), reflecting the increasing allocation of N to mineralization and nitrification.

In the steppe, nitrate accounted for a larger proportion of dissolved N in all horizons (30–36%), although organic N forms still dominated (47–56% of total dissolved N). Ammonium accounted for 8–21%. In contrast to the other ecosystems, we did not find significant changes in the composition of the dissolved N pool between steppe horizons.

## 4 Discussion

In contrast to our hypothesis, we did not find systematic changes in microbial N dynamics between the seven ecosystems studied that would support a decrease in microbial N limitation from arctic over boreal to temperate systems along the 1500 km latitudinal transect. Instead, our findings suggest strong microbial N limitation in organic topsoil horizons of all investigated ecosystems, as indicated by low rates of N mineralization and nitrification in relation to protein depolymerization. In mineral horizons, in contrast, microorganisms maintained significantly higher N mineralization and nitrification rates. Our findings therefore suggest a decrease in microbial N limitation from organic to mineral horizons that was consistent across the range of ecosystems studied.

### 4.1 Differences in Nitrogen Dynamics Between Ecosystems

The seven ecosystems compared in this study cover a wide range of latitudes, climatic conditions, and vegetation types, including sites dominated by grasses, deciduous, and coniferous trees, which differ in litter decomposability [*Cornwell et al.*, 2008] and in their interaction with soil microorganisms. We found that the investigated ecosystems differed strongly in organic topsoil protein depolymerization rates, with highest rates in middle and southern taiga (Figure 3). However, 66% of the variability between sites in protein depolymerization in the organic topsoil was explained by differences in SOM content and microbial biomass (Table3). Including mineral topsoil and subsoil horizons, where no significant differences between ecosystems were observed, SOM content and microbial biomass accounted for 82% of the variability in protein depolymerization rates. Our findings suggest that microbial communities in different ecosystems, and even in different soil horizons, have similar capabilities to release N from proteins.

On a global scale, N limitation of net primary production is expected to decrease with latitude, i.e., from arctic over boreal and temperate to tropical systems [e.g., *Vitousek and Howarth*, 1991], followed by a decrease in the C/N ratio of plant litter inputs, a decrease in the C/N ratio of SOM [*Post et al.*, 1985; *X. Xu et al.*, 2013], and consequently, a decrease in N limitation of soil microorganisms [*Schimel and Bennett*, 2004]. We therefore hypothesized that along the studied transect in western Siberia, SOM C/N ratios would decrease from arctic over boreal to temperate systems, resulting in a decrease in microbial N limitation and a higher microbial allocation of N to mineralization and nitrification. In the organic topsoil, we found a decrease in the C/N ratios of SOM and also of the extractable soil fraction with latitude (Table2 and Table S2 in the supporting information). We did not, however, find indications for a decrease in microbial N limitation. In fact, N mineralization was highest at the taiga sites both related to dry soil and to microbial N (Figure and Figure S2 in the supporting information). Even related to protein depolymerization (that also reached the maximum in the taiga), N mineralization slightly decreased from higher to lower latitudes (Figure 5). In contrast to our findings, *Meyer et al.* [2006] found an increase in N mineralization in organic soil horizons from arctic to temperate ecosystems along a similar transect, thus supporting a decrease in microbial N limitation with decreasing latitude, whereas in a wider range of ecosystems (arctic and antarctic, boreal, temperate, and tropical), *Jones et al.* [2009] did not observe latitudinal patterns in microbial amino acid turnover, pointing at an equal distribution of microbial N limitation across latitudes. Our findings are in line with similar levels of microbial N limitation in arctic, boreal, and temperate ecosystems and suggest that differences in N limitation between these systems were minor compared to differences between soil horizons.

### 4.2 Differences in Nitrogen Dynamics Between Soil Horizons

**Figure 7:**
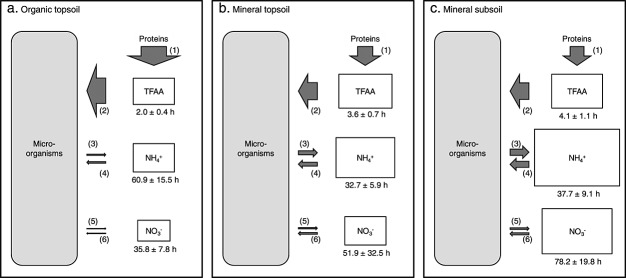
Overview of microbial N dynamics in organic topsoil, mineral topsoil, and mineral subsoil horizons. Areas of boxes are proportional to pool sizes of total free amino acids, ammonium, and nitrate, areas of arrows to hourly gross N fluxes of (1) protein depolymerization, (2) amino acid uptake, (3) N mineralization, (4) ammonium uptake, (5) nitrification, and (6) nitrate uptake. All values are means of the seven study sites and are related to microbial N content, which amounted to 374.1 ± 33.6, 24.6 ± 2.9, and 3.6 ± 0.3 µg N g^−1^ dry soil in organic topsoil, mineral topsoil, and mineral subsoil, respectively (means ± standard errors). The mean residence times of total free amino acids, ammonium, and nitrate (calculated as pool size divided by the average of influx and efflux) are given under the respective boxes (means ± standard errors).

In organic topsoil horizons of all investigated ecosystems, N was efficiently retained by microorganisms and thus likely limited microbial growth (Figures and 4). Microorganisms in mineral topsoil and subsoil horizons, in contrast, maintained higher rates of N mineralization, which in some cases even exceeded protein depolymerization rates. Our findings thus suggest a more open cycling of N in mineral horizons, with higher allocation of N to mineralization and nitrification, higher contributions of ammonium and nitrate to the total dissolved N pool (Figure 6), and higher microbial uptake rates of ammonium and nitrate (Figure S1 in the supporting information) that overall reflect a stronger recycling of N released from microbial cells by mineralization and nitrification and a lower immediate dependence on protein depolymerization as a N source (Figure 7). These findings are supported by previous observations of an increasing contribution of mineral N forms to the total dissolved N pool in taiga soils with depth [*Jones and Kielland*, 2002]. The increasing allocation of N to mineralization thus suggests that microbial communities in mineral soil horizons were not N limited.

Consequently, microorganisms in mineral soil horizons might take up amino acids not to meet their demand for N, but for C. Supporting the role of amino acids as a C source, microbial peptide uptake in mineral soil from a New Zealand grassland site has been found to be controlled by soil C, but not N availability [*Farrell et al.*, 2014]. Also across a range of North American soils, amino acid mineralization was linked to soil C availability, with highest mineralization rates of amino acid C in soils of low C/N and low availability of labile C, suggesting that microorganisms in these soils predominantly used amino acids as an energy source [*McFarland et al.*, 2010].

We hypothesized that microbial N limitation would decrease from organic topsoil to mineral subsoil horizons due to a decrease in the imbalance between the C/N ratios of microbial substrates and microbial biomass, facilitating a higher allocation of N to mineralization and nitrification. Supporting our hypothesis, we found that the C/N imbalance between SOM and the microbial biomass decreased from organic topsoil to mineral topsoil and further to mineral subsoil horizons (Table2) and was significantly correlated with the ratios between gross rates of protein depolymerization, N mineralization, and nitrification, with lower N mineralization and nitrification at high C/N imbalance (i.e., N deficit) and vice versa. However, across all horizons, the C/N imbalance between SOM and the microbial biomass explained only 6–24% of the variability. Although bulk C/N ratios of SOM are frequently used to infer C versus N limitation of the microbial decomposer community, bulk SOM might poorly reflect the substrate actually available for microorganisms—especially in mineral soil horizons, where a large proportion of organic matter is bound to soil minerals (e.g., up to 98% in temperate ecosystems, [*Kögel-Knabner et al.*, 2008]) and thus poorly available for microorganisms [*Kalbitz et al.*, 2005; *Mikutta et al.*, 2007]. The C/N ratio of the extractable soil fraction, which consists of small, soluble compounds that are more easily available for microorganisms, has thus been suggested as a better indicator of soil C versus N availability. However, also the C/N imbalance between the extractable soil fraction and the microbial biomass explained only 0–27% of the microbial N transformation patterns observed in this study (Table4). Our findings therefore suggest that neither bulk SOM C/N nor the extractable C/N strongly reflected the offset between availability and microbial demand for C versus N in the studied soils. In line with these findings, a recent study including both litter and soil samples reported high microbial N use efficiency (the allocation of amino acid N taken up to growth) in litter and poorly decomposed SOM with a high C/N imbalance between organic matter and the microbial biomass but also a high variability in N use efficiency in mineral soil that was poorly explained by the C/N imbalance alone [*Mooshammer et al.*, 2014a].

We therefore suggest two mechanisms that might strongly affect the balance between C and N availability and microbial C and N demand in soils: First, especially in subsoil horizons, C is often bound in compounds of low-energy content [*Rumpel and Kögel-Knabner*, 2011]; microorganisms thus need to invest more energy to gain the same amount of C. Microorganisms in subsoil horizons can therefore be limited in C regardless of C/N ratios, as proposed for a range of ecosystems, including tundra [*Wild et al.*, 2014] and temperate grassland systems [*Fontaine et al.*, 2007]. At our sampling sites, microbial C partitioning between biomass and respiration indeed changed with soil depth, with higher allocation to respiration in subsoil than in topsoil horizons, suggesting a shift in microbial energy demand [*Takriti et al.*, unpublished data]. A low energy content of organic matter might therefore have contributed to C limitation in subsoil horizons. Second, microbial communities adapt to the properties of their substrate. As recently suggested for decomposing leaf litter, microbial decomposer communities might independently regulate the relative turnover rates of C and N, and thus alleviate stoichiometric imbalances between their biomass and the available substrate [*Kaiser et al.*, 2014]. Both mechanisms have the potential to strongly affect the balance between soil C and N availability and microbial C and N demand, and might, especially in subsoil horizons, limit the explanatory power of C/N ratios.

### 4.3 Implications for Ecosystem Nitrogen Cycling

In high-latitude ecosystems, microbial growth and activity is considered to be limited by low N availability [*Schimel and Bennett*, 2004; *Sistla et al.*, 2012]. Although our results support microbial N limitation in organic horizons, microorganisms in mineral horizons maintained high rates of N mineralization and nitrification even in northern ecosystems that indicate microbial N excess. Higher rates of N mineralization and, in particular, nitrification might promote N losses from the ecosystem, e.g., by nitrate leaching or the emission of N_2_O [*Hedin et al.*, 2003]. These fluxes, however, are usually small at high latitudes [*Dise et al.*, 1998; *Kim et al.*, 2013]. Higher microbial N excess in mineral horizons might also indicate higher N availability for plants. This is supported by a recent study showing that in boreal forests, subsoil N contributes significantly to plant N nutrition [*Houle et al.*, 2014].

Nitrogen mineralization is predicted to increase in high latitude ecosystems in the near future as a consequence of higher SOM decomposition rates, and thus higher N release from polymers [*Rustad et al.*, 2001]. Although plant N demand is expected to increase as higher temperatures promote plant primary production [*Natali et al.*, 2012, 2014; *Sistla et al.*, 2013; *L. Xu et al.*, 2013], studies showing an increase in net ecosystem N mineralization with warming suggest an overall positive effect of rising temperatures on soil N availability [*Schimel et al.*, 2004; *Schaeffer et al.*, 2013]. Our findings indicate that such an increase in soil N availability might affect microbial processes especially in organic topsoil horizons, where N is likely limiting microbial growth and activity at least during the growing season. Indeed, in organic topsoils, N availability has been found to control a range of microbial functions, including the amount of extracellular enzymes produced [*Wallenstein et al.*, 2009], the proportions between enzymes targeting different substrates [*Allison et al.*, 2008; *Sistla et al.*, 2012; *Koyama et al.*, 2013], and consequently, rates of organic matter decomposition [*Lavoie et al.*, 2011; *Sistla et al.*, 2012]. In mineral horizons, in contrast, no effect of N addition or even a decrease in decomposition has been observed [*Lavoie et al.*, 2011; *Koyama et al.*, 2013].

Our findings suggest that microbial processes in mineral horizons, even in high-latitude ecosystems such as tundra and boreal forest ecosystems, might be less susceptible to changes in N, but rather in C availability. Also soil C availability is predicted to increase with global warming, since higher plant productivity implies a higher allocation of C from plants to the soil (e.g., as root litter or root exudates). If microbial decomposers in the subsoil are indeed limited in C or energy as indicated by our data, an increase in subsoil C availability might stimulate the decomposition of soil organic matter and promote C losses from these soil horizons, as recently suggested for an Alaskan tundra ecosystem [*Sistla et al.*, 2013].

## 5 Conclusions

Our findings show that even in soils of high latitudes, N limitation of microbial growth is not ubiquitous. Nitrogen was likely limiting in organic soil horizons, as indicated by low N mineralization and nitrification rates in relation to protein depolymerization, but not in mineral horizons, where microbial N demand might have been constrained by low C availability. The microbial demand for C and N drives organic matter decomposition, as well as ecosystem C and N sequestration. Our findings therefore suggest that across all investigated ecosystems, microbial processes and their controls fundamentally changed with soil depth and highlight the need to study the role of deep soil horizons for ecosystem C and N cycling.

## References

[b1] Allison SD, Czimczik CI, Treseder KK (2008). Microbial activity and soil respiration under nitrogen addition in Alaskan boreal forest. Global Change Biol.

[b2] Aulchenko YS, Ripke S, Isaacs A, van Duijn CM (2007). GenABEL: An R library for genome-wide association analysis. Bioinformatics.

[b3] Booth MS, Stark JM, Rastetter E (2005). Controls on nitrogen cycling in terrestrial ecosystems: A synthetic analysis of literature data. Ecol. Monogr.

[b4] Brookes PC, Landman A, Pruden G, Jenkinson DS (1985). Chloroform fumigation and the release of soil nitrogen: A rapid direct extraction method to measure microbial biomass nitrogen in soil. Soil Biol. Biochem.

[b5] Cornwell WK (2008). Plant species traits are the predominant control on litter decomposition rates within biomes worldwide. Ecol. Lett.

[b6] Dise NB, Matzner E, Forsius M (1998). Evaluation of organic horizon C:N ratio as an indicator of nitrate leaching in conifer forests across Europe. Environ. Pollut.

[b7] Farrell M (2013). Oligopeptides represent a preferred source of organic N uptake: A global phenomenon?. Ecosystems.

[b8] Farrell M, Prendergast-Miller M, Jones DL, Hill PW, Condron LM (2014). Soil microbial organic nitrogen uptake is regulated by carbon availability. Soil Biol. Biochem.

[b9] Fontaine S, Barot S, Barré P, Bdioui N, Mary B, Rumpel C (2007). Stability of organic carbon in deep soil layers controlled by fresh carbon supply. Nature.

[b10] Hedin LO, Vitousek PM, Matson PA (2003). Nutrient losses over four million years of tropical forest development. Ecology.

[b11] Hill PW, Farrell M, Jones DL (2012). Bigger may be better in soil N cycling: Does rapid acquisition of small L-peptides by soil microbes dominate fluxes of protein-derived N in soil?. Soil Biol. Biochem.

[b12] Hobbie SE, Schimel JP, Trumbore SE, Randerson JR (2000). Controls over carbon storage and turnover in high-latitude soils. Global Change Biol.

[b13] Hobbie SE, Nadelhoffer KJ, Högberg P (2002). A synthesis: The role of nutrients as constraints on carbon balances in boreal and arctic regions. Plant Soil.

[b14] Houle D, Marty C, Moore J, Ouimet R (2014). Tree species partition N uptake by soil depth in boreal forests. Ecology.

[b15] IUSS Working Group WRB (2007).

[b16] Jones DL, Kielland K (2002). Soil amino acid turnover dominates the nitrogen flux in permafrost-dominated taiga forest soils. Soil Biol. Biochem.

[b17] Jones DL, Owen AG, Farrar JF (2002). Simple method to enable the high resolution determination of total free amino acids in soil solutions and soil extracts. Soil Biol. Biochem.

[b18] Jones DL, Kielland K, Sinclair FL, Dahlgren RA, Newsham KK, Farrar JF, Murphy DV (2009). Soil organic nitrogen mineralization across a global latitudinal gradient. Global Biogeochem. Cycles.

[b19] Kaiser C (2011). Plants control the seasonal dynamics of microbial N cycling in a beech forest soil by belowground C allocation. Ecology.

[b20] Kaiser C, Franklin O, Dieckmann U, Richter A (2014). Microbial community dynamics alleviate stoichiometric constraints during litter decay. Ecol. Lett.

[b21] Kalbitz K, Schwesig D, Rethemeyer J, Matzner E (2005). Stabilization of dissolved organic matter by sorption to the mineral soil. Soil Biol. Biochem.

[b22] Kandeler E, Gerber H (1988). Short-term assay of soil urease activity using colorimetric determination of ammonium. Biol. Fertil. Soils.

[b23] Kielland K, McFarland JW, Ruess RW, Olson K (2007). Rapid cycling of organic nitrogen in taiga forest ecosystems. Ecosystems.

[b24] Kim D, Giltrap D, Hernandez-Ramirez G (2013). Background nitrous oxide emissions in agricultural and natural lands: A metaanalysis. Plant Soil.

[b25] Kögel-Knabner I, Guggenberger G, Kleber M, Kandeler E, Kalbitz K, Scheu S, Eusterhues K, Leinweber P (2008). Organo-mineral associations in temperate soils: Integrating biology, mineralogy, and organic matter chemistry. J. Plant Nutr.

[b26] Koyama A, Wallenstein MD, Simpson RT, Moore JC (2013). Carbon-degrading enzyme activities stimulated by increased nutrient availability in arctic tundra soils. PLoS One.

[b27] Lachouani P, Frank AH, Wanek W (2010). A suite of sensitive chemical methods to determine the *δ*^15^N of ammonium, nitrate and total dissolved N in soil extracts. Rapid Commun. Mass Spectrom.

[b28] Lavoie M, Mack MC, Schuur EAG (2011). Effects of elevated nitrogen and temperature on carbon and nitrogen dynamics in Alaskan arctic and boreal soils. J. Geophys. Res.

[b29] McFarland JW, Ruess RW, Kielland K, Pregitzer K, Hendrick R (2010). Glycine mineralization in situ closely correlates with soil carbon availability across six North American forest ecosystems. Biogeochemistry.

[b30] Meyer H, Kaiser C, Biasi C, Hämmerle R, Rusalimova O, Lashchinsky N, Baranyi C, Daims H, Barsukov P, Richter A (2006). Soil carbon and nitrogen dynamics along a latitudinal transect in western Siberia, Russia. Biogeochemistry.

[b31] Mikutta R, Mikutta C, Kalbitz K, Scheel T, Kaiser K, Jahn R (2007). Biodegradation of forest floor organic matter bound to minerals via different binding mechanisms. Geochim. Cosmochim. Acta.

[b32] Miranda KM, Espey MG, Wink DA (2001). A rapid, simple spectrophotometric method for simultaneous detection of nitrate and nitrite. Nitric Oxide.

[b33] Mooshammer M (2012). Stoichiometric controls of nitrogen and phosphorus cycling in decomposing beech leaf litter. Ecology.

[b34] Mooshammer M (2014a). Adjustment of microbial nitrogen use efficiency to carbon: Nitrogen imbalances regulates soil nitrogen cycling. Nat. Commun.

[b35] Mooshammer M, Wanek W, Zechmeister-Boltenstern S, Richter A (2014b). Stoichiometric imbalances between terrestrial decomposer communities and their resources: Mechanisms and implications of microbial adaptations to their resources. Front. Microbiol.

[b36] Natali SM, Schuur EAG, Rubin RL (2012). Increased plant productivity in Alaskan tundra as a result of experimental warming of soil and permafrost. J. Ecol.

[b37] Natali SM, Schuur EAG, Webb EE, Hicks Pries CE, Crummer KG (2014). Permafrost degradation stimulates carbon loss from experimentally warmed tundra. Ecology.

[b38] Post WM, Pastor J, Zinke PJ, Stangenberger AG (1985). Global patterns of soil nitrogen storage. Nature.

[b39] Prommer J (2014). Biochar decelerates soil organic nitrogen cycling but stimulates soil nitrification in a temperate arable field trial. PLoS One.

[b40] R Development Core Team (2012). R: A Language and Environment for Statistical Computing.

[b41] Rumpel C, Kögel-Knabner I (2011). Deep soil organic matter: A key but poorly understood component of terrestrial C cycle. Plant Soil.

[b42] Rustad LE, Campbell JL, Marion GM, Norby RJ, Mitchell MJ, Hartley AE, Cornelissen JHC, Gurevitch J, GCTE-NEWS (2001). A meta-analysis of the response of soil respiration, net nitrogen mineralization, and aboveground plant growth to experimental ecosystem warming. Oecologia.

[b43] Schaeffer SM, Sharp E, Schimel JP, Welker JM (2013). Soil-plant N processes in a High Arctic ecosystem, NW Greenland are altered by long-term experimental warming and higher rainfall. Global Change Biol.

[b44] Schimel JP, Bennett J (2004). Nitrogen mineralization: Challenges of a changing paradigm. Ecology.

[b45] Schimel JP, Bilbrough C, Welker JM (2004). Increased snow depth affects microbial activity and nitrogen mineralization in two Arctic tundra communities. Soil Biol. Biochem.

[b46] Schnecker J (2015). Microbial community composition shapes enzyme patterns in topsoil and subsoil horizons along a latitudinal transect in western Siberia. Soil Biol. Biochem.

[b47] Shaver GR, Chapin FS (1980). Response to fertilization by various plant growth forms in an Alaskan tundra: Nutrient accumulation and growth. Ecology.

[b48] Sinsabaugh R, Manzoni S, Moorhead DL, Richter A (2013). Carbon use efficiency of microbial communities: Stoichiometry, methodology, and modelling. Ecol. Lett.

[b49] Sistla SA, Asao S, Schimel JP (2012). Detecting microbial N-limitation in tussock tundra soil: Implications for Arctic soil organic carbon cycling. Soil Biol. Biochem.

[b50] Sistla SA, Moore JC, Simpson RT, Gough L, Shaver GR, Schimel JP (2013). Long-term warming restructures Arctic tundra without changing net soil carbon storage. Nature.

[b51] Sterner RW, Elser JJ (2002). Ecological Stoichiometry: The Biology of Elements From Molecules to the Biosphere.

[b52] Stolbovoi V, McCallum I (2002).

[b53] Vitousek PM, Howarth RW (1991). Nitrogen limitation on land and in the sea: How can it occur?. Biogeochemistry.

[b54] Wallenstein MD, McMahon SK, Schimel JP (2009). Seasonal variation in enzyme activities and temperature sensitivities in Arctic tundra soils. Global Change Biol.

[b55] Wanek W, Mooshammer M, Blöchl A, Hanreich A, Richter A (2010). Determination of gross rates of amino acid production and immobilization in decomposing leaf litter by a novel ^15^N isotope pool dilution technique. Soil Biol. Biochem.

[b56] Weidmann NB, Kuse D, Gleditsch KS (2010). The geography of the international system: The CShapes data set. Int. Interact.

[b57] Wild B (2013). Nitrogen dynamics in Turbic Cryosols from Siberia and Greenland. Soil Biol. Biochem.

[b58] Wild B (2014). Input of easily available organic C and N stimulates microbial decomposition of soil organic matter in arctic permafrost soil. Soil Biol. Biochem.

[b59] Xu L (2013). Temperature and vegetation seasonality diminishment over northern lands. Nat. Clim. Change.

[b60] Xu X, Thornton PE, Post WM (2013). A global analysis of soil microbial biomass carbon, nitrogen and phosphorus in terrestrial ecosystems. Global Ecol. Biogeogr.

