# Supplementary material for: Microbial nitrogen dynamics in organic and mineral soil horizons along a latitudinal transect in western Siberia
Source: Global Biogeochem Cycles. 2015 May 12;29(5):567–82. doi: 10.1002/2015GB005084 (PMC4676305; doi:10.1002/2015GB005084)
Supplement: Supplementary file 1 [file gbc0029-0567-sd1.docx]

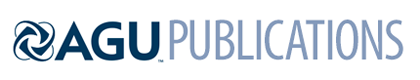


*Global Biogeochemical Cycles*

Supporting Information for

**Microbial nitrogen dynamics in organic and mineral soil horizons along a latitudinal transect in Western Siberia**

Birgit Wild^1,2,3^, Jörg Schnecker^1,2^, Anna Knoltsch^1,2^, Mounir Takriti^1,2^, Maria Mooshammer^1^, Norman Gentsch^4^, Robert Mikutta^4^, Ricardo J. Eloy Alves^2,5^, Antje Gittel^6,7^, Nikolay Lashchinskiy^8^, Andreas Richter^1,2^

^1^ Department of Microbiology and Ecosystem Science, University of Vienna, Vienna, Austria

^2^ Austrian Polar Research Institute, Vienna, Austria

^3^ Department of Earth Sciences, University of Gothenburg, Gothenburg, Sweden

^4^ Institute of Soil Science, Leibniz Universität Hannover, Hannover, Germany

^5^ Department of Ecogenomics and Systems Biology, University of Vienna, Vienna, Austria

^6^ Department of Biology, Centre for Geobiology, University of Bergen, Bergen, Norway

^7^ Department of Bioscience, Center for Geomicrobiology, Aarhus, Denmark

^8^ Central Siberian Botanical Garden, Siberian Branch of Russian Academy of Sciences, Novosibirsk, Russia

**Contents of this file**

Figures S1 to S2

Tables S2, S4, and S5

**Additional Supporting Information (Files uploaded separately)**

Captions for Tables S1 and S3

**Introduction**

Figure S1 shows gross rates of microbial amino acid, ammonium, and nitrate uptake. Figure S2 displays gross rates of protein depolymerization, N mineralization, and nitrification related to microbial N, and thus complements Figure 3, where gross rates are shown on a dry soil basis. Tables S1, S2 and S3 provide additional statistics: Table S1 shows significant differences in basic parameters between different horizons of the seven investigated ecosystems, and across all sites. Table S2 shows significant differences in stoichiometric parameters between different ecosystems for each horizon class. Table S3 shows significant differences in gross N transformation rates, related to dry soil or microbial N, as well as of ratios between individual rates, between different horizons of the seven investigated ecosystems, and across all sites. Table S4 provides concentrations of dissolved N pools in the studied samples, and Table S5 shows significant differences in the composition of the total dissolved N pool between soil horizons.

Figure S1. Gross rates of microbial amino acid, ammonium, and nitrate uptake in organic topsoil (Org. Top.), mineral topsoil (Min. Top.), and mineral subsoil (Min. Sub.), related to dry soil (a-c) and microbial N (N_mic_; d-f). Bars represent means with standard errors across the seven ecosystems studied; different letters indicate significant differences between horizons. Note the differences in scaling.

Figure S2. Gross rates of protein depolymerization, N mineralization, and nitrification, related to microbial N (N_mic_), in three soil horizons of seven ecosystems along a latitudinal transect in Western Siberia. All bars represent means with standard errors, different letters indicate significant differences between sites for each horizon. N. Taiga, northern taiga; M. Taiga, middle taiga; S. Taiga, southern taiga; FS, forest steppe.

Table S1. Significance of differences in basic parameters between soil horizons (p < 0.05). Corresponding data can be found in Table 2.

| **Table S2.** Significance of differences in stoichiometric parameters between ecosystems for individual soil horizons, with different letters indicating significant differences at p < 0.05^a^ | | | | | | | | | | | | | | | | | | | |
| --- | --- | --- | --- | --- | --- | --- | --- | --- | --- | --- | --- | --- | --- | --- | --- | --- | --- | --- | --- |
|  | C/N_SOM_ | | |  | C/N_extr_ | | |  | C/N_mic_ | | |  | Imbalance_SOM_^b^ | | |  | Imbalance_extr_^b^ | | |
|  | Org.  Top. | Min.  Top. | Min.  Sub. |  | Org.  Top. | Min.  Top. | Min.  Sub. |  | Org.  Top. | Min.  Top. | Min.  Sub. |  | Org.  Top. | Min.  Top. | Min.  Sub. |  | Org.  Top. | Min.  Top. | Min.  Sub. |
| Tundra | a | bc | bcd |  | a | abc | abc |  | a | cd | b |  | a | ab | a |  | a | a | a |
| Northern taiga | a | a | a |  | ab | a | a |  | b | a | a |  | a | a | a |  | ab | ab | b |
| Middle taiga | b | ab | a |  | ab | bc | bc |  | b | bcd | ab |  | b | a | a |  | b | abc | b |
| Southern taiga | b | cd | cd |  | bc | c | bc |  | bc | d | bc |  | a | ab | a |  | ab | b | b |
| Forest steppe: Forest | c | d | bc |  | bc | c | bc |  | bc | abc | bc |  | c | c | a |  | b | c | ab |
| Forest steppe: Meadow | d | cd | b |  | bc | ab | bc |  | bc | abc | abc |  | c | bc | a |  | b | bc | b |
| Steppe | e | e | d |  | c | abc | c |  | c | ab | b |  | d | c | a |  | c | bc | b |
| ^a^Org. Top., organic topsoil; Min. Top., mineral topsoil; Min. Sub., mineral subsoil; C/N_SOM_, SOM C/N; C/N_extr_, C/N of the extractable soil fraction; C/N_mic_, C/N of the microbial biomass  ^b^Imbalance_SOM_ was calculated as the ratio of C/N_SOM_ over C/N_mic_, Imbalance_extr_ as the ratio of C/N_extr_ over C/N_mic_ | | | | | | | | | | | | | | | | | | | |

Table S2. Significance of differences in stoichiometric parameters between ecosystems for individual soil horizons (p < 0.05). Corresponding data can be found in Table 2.

Table S3. Significance of differences in N transformation rates between soil horizons (p < 0.05). Corresponding data are shown in Figures 3, 4, and S2.

| **Table S4.** Concentrations of dissolved N pools^a^ | | | | | | | | | | | | | | |
| --- | --- | --- | --- | --- | --- | --- | --- | --- | --- | --- | --- | --- | --- | --- |
|  | TDN^b^  (µg N g^-1^ dry soil) | |  | DON^b^  (µg N g^-1^ dry soil) | |  | TFAA^b^  (µg N g^-1^ dry soil) | |  | Ammonium  (µg N g^-1^ dry soil) | |  | Nitrate  (µg N g^-1^ dry soil) | |
| Tundra |  |  |  |  |  |  |  |  |  |  |  |  |  |  |
| Organic topsoil | 56.16 | (7.09) |  | 53.79 | (6.87) |  | 5.31 | (0.51) |  | 1.84 | (0.34) |  | 0.52 | (0.13) |
| Mineral topsoil | 4.64 | (0.90) |  | 3.67 | (0.80) |  | 0.61 | (0.11) |  | 0.81 | (0.18) |  | 0.16 | (0.09) |
| Mineral subsoil | 0.20 | (0.15) |  | 0.02 | (0.15) |  | 0.04 | (0.01) |  | 0.11 | (0.04) |  | 0.08 | (0.05) |
| Northern taiga |  |  |  |  |  |  |  |  |  |  |  |  |  |  |
| Organic topsoil | 137.71 | (11.43) |  | 132.95 | (11.08) |  | 13.14 | (0.69) |  | 4.29 | (0.46) |  | 0.48 | (0.17) |
| Mineral topsoil | 9.94 | (0.93) |  | 8.95 | (0.82) |  | 1.19 | (0.14) |  | 0.78 | (0.29) |  | 0.21 | (0.13) |
| Mineral subsoil | 3.06 | (0.63) |  | 2.28 | (0.46) |  | 0.23 | (0.02) |  | 0.57 | (0.28) |  | 0.21 | (0.13) |
| Middle taiga |  |  |  |  |  |  |  |  |  |  |  |  |  |  |
| Organic topsoil | 184.50 | (21.77) |  | 175.12 | (20.12) |  | 24.14 | (2.28) |  | 8.92 | (1.62) |  | 0.46 | (0.07) |
| Mineral topsoil | 30.42 | (9.22) |  | 28.49 | (9.15) |  | 3.71 | (1.01) |  | 1.82 | (0.39) |  | 0.12 | (0.03) |
| Mineral subsoil | 6.58 | (1.85) |  | 4.90 | (1.47) |  | 0.41 | (0.10) |  | 1.40 | (0.48) |  | 0.28 | (0.11) |
| Southern taiga |  |  |  |  |  |  |  |  |  |  |  |  |  |  |
| Organic topsoil | 416.31 | (90.14) |  | 354.41 | (62.25) |  | 25.79 | (7.83) |  | 58.35 | (28.37) |  | 3.55 | (2.19) |
| Mineral topsoil | 17.16 | (2.90) |  | 13.88 | (1.50) |  | 1.65 | (0.19) |  | 2.58 | (1.07) |  | 0.71 | (0.43) |
| Mineral subsoil | 2.49 | (0.39) |  | 1.53 | (0.33) |  | 0.11 | (0.01) |  | 0.69 | (0.19) |  | 0.28 | (0.07) |
| Forest steppe: Forest |  |  |  |  |  |  |  |  |  |  |  |  |  |  |
| Organic topsoil | 116.39 | (45.06) |  | 101.43 | (38.40) |  | 15.20 | (5.64) |  | 14.58 | (6.82) |  | 0.37 | (0.07) |
| Mineral topsoil | 7.91 | (0.80) |  | 6.40 | (0.67) |  | 0.66 | (0.14) |  | 0.86 | (0.28) |  | 0.65 | (0.21) |
| Mineral subsoil | 1.92 | (0.43) |  | 1.51 | (0.38) |  | 0.09 | (0.01) |  | 0.23 | (0.03) |  | 0.18 | (0.06) |
| Forest steppe: Meadow |  |  |  |  |  |  |  |  |  |  |  |  |  |  |
| Organic topsoil | 85.51 | (17.01) |  | 78.79 | (15.63) |  | 9.23 | (2.24) |  | 6.25 | (1.54) |  | 0.46 | (0.15) |
| Mineral topsoil | 6.47 | (0.54) |  | 5.55 | (0.62) |  | 0.65 | (0.08) |  | 0.74 | (0.37) |  | 0.18 | (0.13) |
| Mineral subsoil | 1.50 | (0.24) |  | 1.03 | (0.23) |  | 0.25 | (0.13) |  | 0.27 | (0.07) |  | 0.19 | (0.11) |
| Steppe |  |  |  |  |  |  |  |  |  |  |  |  |  |  |
| Organic topsoil | 11.80 | (3.21) |  | 5.99 | (0.91) |  | 0.42 | (0.05) |  | 0.91 | (0.32) |  | 4.91 | (2.47) |
| Mineral topsoil | 7.41 | (1.10) |  | 3.43 | (0.86) |  | 0.33 | (0.07) |  | 1.67 | (0.46) |  | 2.31 | (0.80) |
| Mineral subsoil | 3.64 | (0.32) |  | 2.29 | (0.35) |  | 0.08 | (0.01) |  | 0.31 | (0.11) |  | 1.04 | (0.16) |
| ^a^All values are means with standard errors in brackets.  ^b^TDN, total dissolved N; DON, dissolved organic N; TFAA, total free amino acids. | | | | | | | | | | | | | | |

Table S4. Concentrations of dissolved N pools. See Figure 6 for contributions of individual pools to total dissolved N.

| **Table S5.** Significance of differences in the composition of the dissolved N pool between horizons, with different letters indicating significant differences at p < 0.05^a^ | | | | | | | | | | | | | | | |
| --- | --- | --- | --- | --- | --- | --- | --- | --- | --- | --- | --- | --- | --- | --- | --- |
|  | DON  (% of TDN) | | |  | TFAA  (% of TDN) | | |  | Ammonium  (% of TDN) | | |  | Nitrate  (% of TDN) | | |
|  | Org.  Top. | Min.  Top. | Min.  Sub. |  | Org.  Top. | Min.  Top. | Min.  Sub. |  | Org.  Top. | Min.  Top. | Min.  Sub. |  | Org.  Top. | Min.  Top. | Min.  Sub. |
| Tundra | a | b | b |  | b | a | ab |  | b | a | a |  | a | a | a |
| Northern taiga | a | b | b |  | a | a | a |  | b | ab | a |  | a | a | a |
| Middle taiga | a | a | b |  | a | a | b |  | b | b | a |  | a | a | a |
| Southern taiga | a | a | b |  | a | a | a |  | b | ab | a |  | b | ab | a |
| Forest steppe: Forest | a | ab | b |  | a | b | b |  | a | a | a |  | b | a | a |
| Forest steppe: Meadow | a | a | a |  | a | a | a |  | a | a | a |  | b | b | a |
| Steppe | a | a | a |  | a | a | a |  | a | a | a |  | a | a | a |
| All sites | a | b | c |  | a | a | b |  | c | b | a |  | c | b | a |
| ^a^Org. Top., organic topsoil; Min. Top., mineral topsoil; Min. Sub., mineral subsoil; TDN, total dissolved N; DON, dissolved organic N; TFAA, total free amino acids | | | | | | | | | | | | | | | |

Table S5. Significance of differences in the composition of the total dissolved N pool between soil horizons (p < 0.05). Corresponding data are shown in Figure 6.
