# Supplementary material for: Microbial nitrogen dynamics in organic and mineral soil horizons along a latitudinal transect in western Siberia
Source: Global Biogeochem Cycles. 2015 May 12;29(5):567–82. doi: 10.1002/2015GB005084 (PMC4676305; doi:10.1002/2015GB005084)
Supplement: Supplementary file 2 [file gbc0029-0567-sd2.docx]

| **Table S1.** Significance of differences in basic parameters between soil horizons, with different letters indicating significant differences at p < 0.05^a^ | | | | | | | | | | | | | | | | | | | | | | | | | | | | | | | | | | | | | | | |
| --- | --- | --- | --- | --- | --- | --- | --- | --- | --- | --- | --- | --- | --- | --- | --- | --- | --- | --- | --- | --- | --- | --- | --- | --- | --- | --- | --- | --- | --- | --- | --- | --- | --- | --- | --- | --- | --- | --- | --- |
|  | C  (mg g^-1^ d.s.) | | |  | N  (mg g^-1^ d.s.l) | | |  | Microbial C  (µg g^-1^ d.s.) | | |  | Microbial N  (µg g^-1^ d.s.) | | |  | C/N_SOM_ | | |  | C/N_extr_ | | |  | C/N_mic_ | | |  | Imbalance_SOM_^b^ | | |  | Imbalance_extr_^b^ | | |  | pH | | |
|  | Org.  Top. | Min.  Top. | Min.  Sub. |  | Org.  Top. | Min.  Top. | Min.  Sub. |  | Org.  Top. | Min.  Top. | Min.  Sub. |  | Org.  Top. | Min.  Top. | Min.  Sub. |  | Org.  Top. | Min.  Top. | Min.  Sub. |  | Org.  Top. | Min.  Top. | Min.  Sub. |  | Org.  Top. | Min.  Top. | Min.  Sub. |  | Org.  Top. | Min.  Top. | Min.  Sub. |  | Org.  Top. | Min.  Top. | Min.  Sub. |  | Org.  Top. | Min.  Top. | Min.  Sub. |
| Tundra | a | b | c |  | a | b | c |  | a | b | c |  | a | b | c |  | a | b | c |  | a | a | a |  | c | b | a |  | a | b | c |  | a | a | a |  | a | a | a |
| Northern taiga | a | b | c |  | a | b | c |  | a | b | b |  | a | b | c |  | a | b | c |  | b | b | a |  | c | b | a |  | a | b | c |  | a | b | b |  | c | b | a |
| Middle taiga | a | b | c |  | a | b | c |  | a | b | c |  | a | b | c |  | a | ab | b |  | b | c | a |  | c | b | a |  | a | b | c |  | a | b | b |  | a | b | ab |
| Southern taiga | a | b | c |  | a | b | c |  | a | b | c |  | a | b | c |  | a | b | c |  | ab | b | a |  | c | b | a |  | a | b | c |  | a | b | b |  | a | b | b |
| Forest steppe: Forest | a | b | c |  | a | b | c |  | a | b | c |  | a | b | c |  | a | b | c |  | b | b | a |  | c | b | a |  | a | b | c |  | a | c | b |  | a | b | c |
| Forest steppe: Meadow | a | b | c |  | a | b | c |  | a | b | c |  | a | b | c |  | a | b | c |  | b | b | a |  | c | b | a |  | a | b | c |  | a | b | b |  | a | b | b |
| Steppe | a | b | c |  | a | b | c |  | a | a | b |  | a | b | c |  | a | a | b |  | c | b | a |  | b | a | a |  | a | b | c |  | a | a | a |  | b | b | a |
| All sites | a | b | c |  | a | b | c |  | a | b | c |  | a | b | c |  | a | b | c |  | b | b | a |  | c | b | a |  | a | b | c |  | a | b | b |  | a | b | ab |
| ^a^Org. Top., organic topsoil; Min. Top., mineral topsoil; Min. Sub., mineral subsoil; d.s., dry soil; C/N_SOM_, SOM C/N; C/N_extr_, C/N of the extractable soil fraction; C/N_mic_, C/N of the microbial biomass  ^b^Imbalance_SOM_ was calculated as the ratio of C/N_SOM_ over C/N_mic_, Imbalance_extr_ as the ratio of C/N_extr_ over C/N_mic_ | | | | | | | | | | | | | | | | | | | | | | | | | | | | | | | | | | | | | | | |
