# Supplementary material for: Microbial nitrogen dynamics in organic and mineral soil horizons along a latitudinal transect in western Siberia
Source: Global Biogeochem Cycles. 2015 May 12;29(5):567–82. doi: 10.1002/2015GB005084 (PMC4676305; doi:10.1002/2015GB005084)
Supplement: Supplementary file 3 [file gbc0029-0567-sd3.docx]

| **Table S3.** Significance of differences in N dynamics between soil horizons, with different letters indicating significant differences at p < 0.05^a^ | | | | | | | | | | | | | | | | | | | | | | | | | | | | | | | | | | | |
| --- | --- | --- | --- | --- | --- | --- | --- | --- | --- | --- | --- | --- | --- | --- | --- | --- | --- | --- | --- | --- | --- | --- | --- | --- | --- | --- | --- | --- | --- | --- | --- | --- | --- | --- | --- |
|  | Protein depol.^b^  (µg N g^-1^ d.s. h^-1^) | | |  | N mineralization^b^  (µg N g^-1^ d.s. h^-1^) | | |  | Nitrification^b^  (µg N g^-1^ d.s. h^-1^) | | |  | Protein depol.^b^  (mg N g^-1^ N_mic_ h^-1^) | | |  | N mineralization^b^  (mg N g^-1^ N_mic_ h^-1^) | | |  | Nitrification^b^  (mg N g^-1^ N_mic_ h^-1^) | | |  | N mineralization/  protein depol. | | |  | Nitrification/  protein depol. | | |  | Nitrification/  N mineralization | | |
|  | Org.  Top. | Min.  Top. | Min.  Sub. |  | Org.  Top. | Min.  Top. | Min.  Sub. |  | Org.  Top. | Min.  Top. | Min.  Sub. |  | Org.  Top. | Min.  Top. | Min.  Sub. |  | Org.  Top. | Min.  Top. | Min.  Sub. |  | Org.  Top. | Min.  Top. | Min.  Sub. |  | Org.  Top. | Min.  Top. | Min.  Sub. |  | Org.  Top. | Min.  Top. | Min.  Sub. |  | Org.  Top. | Min.  Top. | Min.  Sub. |
| Tundra | a | b | b |  | n.a. | a | a |  | a | a | a |  | a | a | a |  | n.a. | b | a |  | b | a | a |  | n.a. | b | a |  | b | a | a |  | n.a. | a | a |
| Northern taiga | a | b | b |  | a | b | c |  | a | ab | b |  | a | a | a |  | b | a | a |  | a | a | a |  | a | a | a |  | a | a | a |  | a | a | a |
| Middle taiga | a | b | n.a. |  | a | b | n.a. |  | a | a | a |  | a | a | n.a. |  | a | a | n.a. |  | a | a | a |  | a | a | n.a. |  | a | n.a. | n.a. |  | a | a | n.a. |
| Southern taiga | a | b | c |  | a | b | c |  | a | ab | b |  | a | b | ab |  | a | a | a |  | a | a | a |  | b | b | a |  | a | a | a |  | b | ab | a |
| Forest steppe: Forest | a | b | b |  | a | b | b |  | a | n.a. | b |  | a | a | a |  | a | a | a |  | b | n.a. | a |  | a | a | a |  | a | a | a |  | a | n.a. | a |
| Forest steppe: Meadow | a | b | c |  | a | ab | b |  | a | a | b |  | a | a | a |  | a | a | a |  | b | a | a |  | b | a | a |  | b | a | a |  | a | a | a |
| Steppe | a | a | b |  | ab | a | b |  | a | ab | b |  | a | a | a |  | b | a | ab |  | a | a | a |  | a | a | a |  | a | a | a |  | a | b | ab |
| All sites | a | b | c |  | a | b | c |  | a | b | c |  | a | ab | b |  | b | a | a |  | b | a | a |  | b | a | a |  | b | a | a |  | a | a | a |
| ^a^Org. Top., organic topsoil; Min. Top., mineral topsoil; Min. Sub., mineral subsoil  ^b^Gross rates of protein depolymerization (Protein depol.), N mineralization and nitrification, related to dry soil (d.s.) or to microbial N (N_mic_) | | | | | | | | | | | | | | | | | | | | | | | | | | | | | | | | | | | |
